# Synthesis of porous carbon material based on biomass derived from hibiscus sabdariffa fruits as active electrodes for high-performance symmetric supercapacitors

**DOI:** 10.1039/d0ra09509e

**Published:** 2020-12-23

**Authors:** Hamouda Adam Hamouda, Shuzhen Cui, Xiuwen Dai, Lele Xiao, Xuan Xie, Hui Peng, Guofu Ma

**Affiliations:** Key Laboratory of Eco-Environment-Related Polymer Materials of Ministry of Education, Key Laboratory of Polymer Materials of Gansu Province, College of Chemistry and Chemical Engineering, Northwest Normal University Lanzhou 730070 China magf@nwnu.edu.cn; Department of Chemistry, Faculty of Science, University of Kordofan El Obeid 51111 Sudan

## Abstract

Carbon-based materials are manufactured as high-performance electrodes using biomass waste in the renewable energy storage field. Herein, four types of hierarchical porous activated carbon using hibiscus sabdariffa fruits (HBFs) as a low-cost biomass precursor are synthesized through carbonization and activation. NH_4_Cl is used as a chemical blowing agent to form carbon nanosheets, which are the first types of hibiscus sabdariffa fruit-based carbon (HBFC-1) sample, and KOH also forms a significant bond in the activation process. The prepared HBFC-1 is chosen to manufacture the symmetric supercapacitor due to its rough surface and high surface area (1720.46 m^2^ g^−1^), making it show a high specific capacity of 194.50 F g^−1^ at a current density of 0.5 A g^−1^ in a three-electrode system. Moreover, the HBFC-1 based symmetric supercapacitor devices display a high energy density of 13.10 W h kg^−1^ at a power density of 225.00 W kg^−1^, and a high specific capacity of 29 F g^−1^ at 0.5 A g^−1^. Additionally, excellent cycle life is observed (about 96% of capacitance retained after 5000 cycles). Therefore, biomass waste, especially hibiscus sabdariffa fruit based porous carbon, can be used as the electrode for high-performance supercapacitor devices.

## Abstract 2

Carbon-based materials are manufactured as high-performance electrodes using biomass waste in the renewable energy storage field.

## Graphical abstract

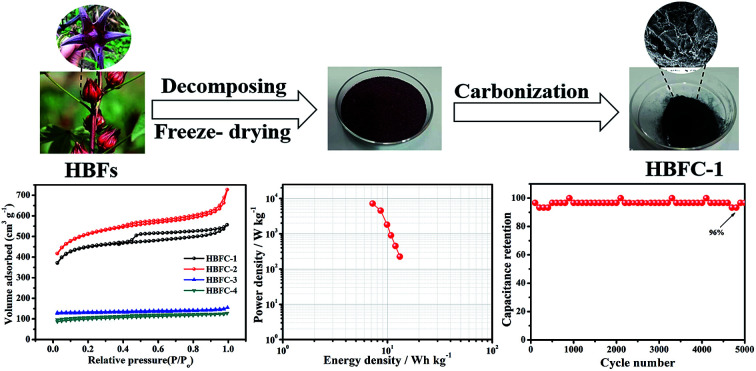

## 1. Introduction

With the rapid social and economic development, the energy crisis and environmental problems have made it essential to develop eco-friendly and low-cost energy sources.^1,2^ Therefore, renewable energy sources with high power/energy densities have much inspired widespread research interests to develop high-performance energy storage devices.^3^ Among the different electrical energy storage devices, supercapacitors have received great attraction due to their unique advantages of low-cost, excellent cycle stability, fast charge–discharge rates, and high power densities.^4,5^ However, the supercapacitor has a lower energy density than batteries, which affects its application.^6^ Recently, to overcome environmental problems and manufacture low-cost and high-performance supercapacitors, the use of biomass waste to prepare porous carbon electrodes has become an important new strategy. There are various, actions for biomass waste-based activated carbon with a large variety of structural features appropriate for supercapacitors' manufacture.^7,8^ However, cost-effectiveness still depends on typical activations.^9^ The porous activated carbons derived from different biomass precursors have different electrochemical performances as supercapacitor electrodes due to their different microstructures, pore size distribution, and dopant heteroatoms. Therefore, selecting the convenient heteroatom-containing biomass precursor is very important, and the effect of the pore structure plays an important role in amelioration electrochemical performance in aqueous electrolytes.^10^ The properties of the biomass-derived activated porous carbons, including surface chemistry, are affected by the quality of the material and porosity, pore frame, pore size distribution, and the activation conditions.^11,12^ To utilize porous carbons in energy storage equipment, the optimizing their structural characteristics is of great significance to practical applications. For their use as electrodes in supercapacitors, these materials need to possess a high specific surface area simultaneously with a suitable pore size distribution that combines narrow micropores appropriate for the accommodation of ions with super pores that favor ion transport and serve for storing electrolyte.^13^ There are two activation techniques for the derivation of activated carbons from biomass precursor's physical and chemical activations. In a chemical activation process, activated carbons are produced through the mixing of the precursor with an activating the strong base agent such as KOH and chemical-blowing agent NH_4_Cl the subsequent pyrolysis under suitable conditions in an inert atmosphere.^14^ Physical activation can involve the carbonization of materials at high temperatures (600–900 °C) under the air.^15^ Compared with chemical activation, physical activation has many downsides, including high activation temperature and long activation time. It can be completed in a several-steps activation process with possibly obtaining less specific surface area activated carbons than chemical activation.^16–18^ Diverse biomass precursors, such as fish scale,^19^ fallen leaves,^20^ ginkgo shells,^21^ willow catkins,^22^ water bamboo,^23^ yeast cells,^24^ pig bone,^25^ raw cotton,^26^ cherry stone,^27^ waste tea-leaves,^28^ pine-cone,^29^ cow dung,^30^ waste paper,^31^ human hair,^32^ baobab fruitshells,^33^ sewage sludge,^34^ and wild jujube pits,^35^ these natural materials have been chemically converted into active carbon for supercapacitors because of their rich source, inexpensive and unique structure. The Hibiscus sabdariffa (Roselle) is a plant with beautiful red flowers grown for production and sometimes for decoration that grows in El-Debibat City, Kordofan State, Sudan, and other parts of the word. The hibiscus flower is used in many industries such as food, medical drugs, cosmetics, and others,^36^ but the hibiscus sabdariffa fruits (HBFs) belongs to agricultural waste, has low cost and no commercial value. Here, we synthesized types of hierarchical 3D porous structure activated carbons derived from Hibiscus sabdariffa fruits (HBFs) as a low-cost biomass precursor through pyrolysis processes by using NH_4_Cl and KOH as activating agent. The porous activated carbons (HBFC-1) can be used as high-performance symmetric supercapacitor electrodes.

## 2. Experimental

### 2.1. Materials

Hibiscus sabdariffa fruits (collected from El-Debibat region, Kordofan State, Sudan). Ammonium chloride (NH_4_Cl, Aladdin Co., Ltd., China), Nickel foam, potassium hydroxide (KOH, Sinopharm Chemical Reagent Co., Ltd., China), Hydrochloric acid (HCl, Aladdin Ltd., Shanghai, China), polyvinylidene fluoride (PVDF, Aladdin Ltd., Shanghai, China) and carbon black (CB, Aladdin Ltd., Shanghai, China), *N*-methyl pyrrolidinone solution (NMP, Aladdin Ltd., Shanghai, China). All chemicals were commercially available and employed without further purification.

### 2.2. Preparation of hibiscus sabdariffa fruits derived activated carbon (HBFC)

The hibiscus sabdariffa fruits (HBF) are the pieces volume about (1–2 cm^3^), were first fragmented into small pieces, washed several times with distilled water and absolute ethanol with drying in an oven at 60 °C for a long time about 18 h. Typically, 6 g of dried HBF is immersed in 60 mL distilled water for 3 h. Then, the suspended solution of the sample was transferred into 100 mL Teflon-lined stainless autoclave and incubated for 10 h at 120 °C. After that, cooled down to room temperature followed by ultrasonic treatment for 2 h to form HBF a homogenous solution. The hibiscus solution was frozen-dried with the adding 3 g NH_4_Cl as a chemical-blowing agent. We preferred that the carbonization process was performed at 800 °C for 2 h in a tube furnace in N_2_ at a rate of 5 °C min^−1^ to carbon powder. For furthermore activation process, the previous carbon powder was mixed with a KOH (1 : 1) in 10 mL distilled water. Then, the treated carbon aerogel was dried at 120 °C and heated up under N_2_ at 800 °C for 3 h with a heating rate of 5 °C min^−1^. After that, cooled down to room temperature, the obtained activated carbon powder was washed several times with HCl (diluted) and distilled water. The activated carbon was obtained after drying at 60 °C overnight and denoted as HBFC-1 (the yield of HBFC-1 is about 58.3%). For comparison, activated carbon was prepared *via* the above same process without NH_4_Cl and with KOH activation denoted as HBFC-2, prepared with NH_4_Cl and without KOH denoted as HBFC-3, prepared without NH_4_Cl and KOH denoted as HBFC-4.

### 2.3. Materials characterizations

The structures and morphologies of the HBFCs samples were characterized by scanning electron microscopy (FE-SEM, Carl Zeiss Ultra Plus, and Germany) at an acceleration voltage of 5 kV, and the transmission electron microscopy (TEM, JEM-1200EX, Japan). X-ray diffraction (XRD) was conducted using a Rigaku D/Max-2400 diffractometer equipped with Cu Kα radiation (*k* = 1.5418 Å). Raman spectra were recorded through a *Via* Raman spectrometer (Renishaw) with an Argon ion laser (*λ* = 514.5 nm) at ambient temperature. The Brunauer–Emmett–Teller surface area (*S*_BET_) and pore structure of the carbon samples were analyzed by nitrogen adsorption in a Micromeritics ASAP 2020 nitrogen adsorption apparatus (U.S.A.), and all samples were degassed at 200 °C before nitrogen adsorption measurements. X-ray photoelectron spectroscopy (XPS) measurement was performed on an Escalab 210 system (Germany).

### 2.4. Surface wetting analysis

Water (individual drop) the volume is 5 μL was used to measure the wettability of the surface equipped as water contact angle measurements of the SL200KB at 20 °C.

### 2.5. Electrochemical measurements

The electrochemical performances of all as-prepared electrode materials (HBFCs) were studied by cyclic voltammetry (CV), galvanostatic charge–discharge (GCD), and electrical impedance spectroscopy (EIS) measurements with an electrochemical workstation (CHI 660D). And the cycle-life stability was performed on the cycling testing equipment (CT2001A, Wuhan Land Electronics Co. Ltd., China).

#### 2.5.1. Three-electrode system

The electrochemical a three-electrode system, the as-prepared HBFCs (1 × 1 cm^2^) as active materials, Hg/HgO as the reference electrode, and Pt wire as a counter electrode, in a 2 M KOH solution at room temperature. The working electrode was prepared *via* mixing the as-prepared activated carbon samples, carbon black, and polyvinylidene fluoride (PVDF) with a mass ratio of 8 : 1 : 1 in some drop *N*-methyl pyrrolidinone solution to form slurry. The Ni foam (1.0 cm^2^) and dried at 60 °C for 24 h (Nickel foam is used as a current collector), then weighted and pressed into sheets under 15 MPa. The total mass of each electrode was limited to vary from 3.0 to 5.0 mg. After that, the three-electrode system was tested in the 2 M KOH aqua electrolyte. The CV and GCD measurements were realized at different scan rates (10–200 mV s^−1^) and current densities (0.5–20 A g^−1^) in the potential window of (−1 to 0 V). The specific capacitance of all as-prepared working electrodes were calculated from the GCD curves according to the eqn (1) and (2) below.

1 *C*_m_ = *I*Δ*t*/(*m*Δ*V*)

2 *C*_s_ = *I*Δ*t*/(*S*Δ*V*)

Where the *C*_m_ (F g^−1^) and *C*_s_ (mF cm^−2^) are the specific and area capacitances, *I* (A) is the discharge current, Δ*t* (s) is discharge time, *m* (g) is the weight of the active material, and Δ*V* (V) is the potential window, for the three-electrode system.

#### 2.5.2. Two-electrode system

The electrochemical performance of the HBFC-1//HBFC-1 device was realized in a two-electrode electrochemical system configuration. The working electrodes were prepared also *via* mixing the as-prepared activated carbon samples, carbon black, and polyvinylidene fluoride (PVDF) with a mass ratio of 8 : 1 : 1 in some drop for *N*-methyl pyrrolidinone solution to form slurry. The HBFC-1 electrode fitted with the separator and electrolyte solution were symmetrically assembled into sandwich-type cell construction (electrode/separator/electrode). The specific capacitance of the as-assembled, all-solid-state supercapacitor device was calculated from the GCD curves according to eqn (1) the energy density and power density for a supercapacitor cell were also calculated according to the following eqn (3) and (4).^37^

3 *E* = *C*_m_ × Δ*V*^2^/2

4 *P* = *E*/Δ*t*

where the *C*_m_ is the specific capacitance of the cell (F g^−1^), Δ*V* is the voltage change during the discharge process (V), Δ*t* is discharge time (s), P is the power density (W kg^−1^), and *E* is the energy density (W h kg^−1^).

## 3. Results and discussion

### 3.1. Mechanism of the HBFCs formation

**Scheme 1:**
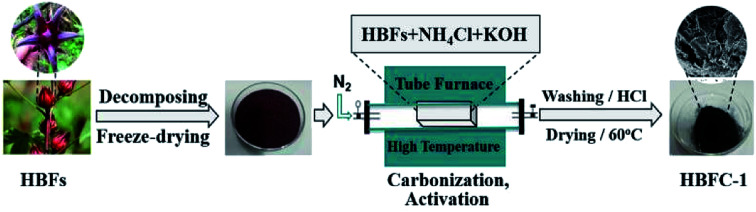
Schematic of the preparation process of porous carbon materials (HBFC-1).

The schematic illustration for the preparation of HBFC-1 is described in Scheme 1. The process mainly consists of two important steps, the hydrothermal process, and the carbonization process. In the hydrothermal process, the hibiscus can be carbonized incompletely and formed carbon aerogels. And in the carbonization process, under the influence of NH_4_Cl and/or KOH, the carbon aerogels became porous carbon completely. The KOH used as an activator can enhance the HBFCs (HBFC-1, HBFC-2) pore size, while the NH_4_Cl is a chemical-blowing agent for the preparation of HBFCs which can significantly enhance their pore volume and provide effective nitrogen doping in (HBFC-1 and HBFC-2) materials.

### 3.2. Morphology and structure characterization

**Fig. 1:**
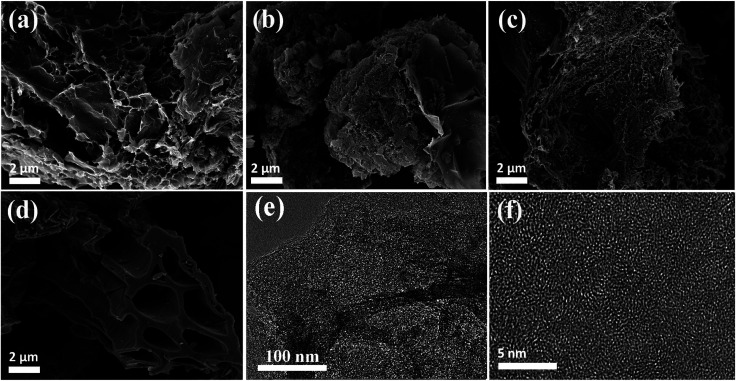
SEM images of (a) HBFC-1; (b) HBFC-2; (c) HBFC-3; (d) HBFC-4; (e) TEM images of HBFC-1 and; (f) HR-TEM image of HBFC-1.

**Fig. 2:**
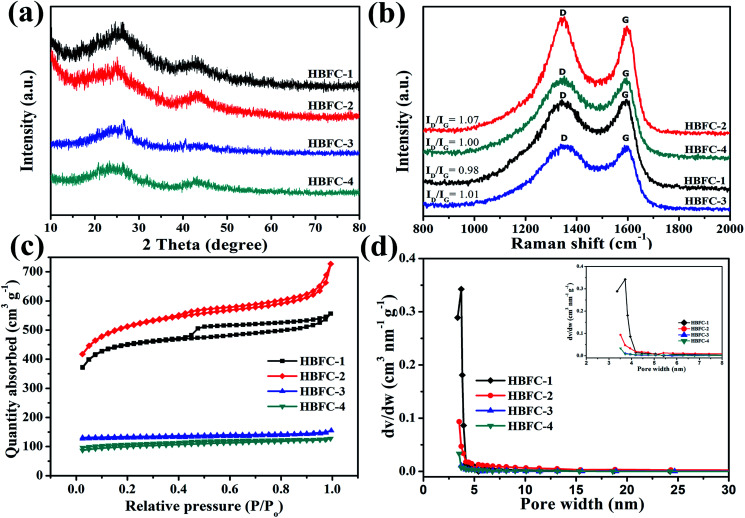
(a) XRD patterns of HBFC-1, HBFC-2, HBFC-3, and HBFC-4; (b) Raman spectrum of the samples; (c) nitrogen sorption isotherms; and (d) the pore size distribution and partial enlargement (inset).

The morphologies and microstructures of the samples (HBFs, HBFC-1, HBFC-2, HBFC-3, and HBFC-4) were characterized by scanning electron microscope (SEM) images. According to Fig. 1a, the HBFC-1 surface illustrates an interconnected and twisted structure with formed carbon layers accumulated randomly and macropores in the process. This morphology can owe to the activating treatment of both NH_4_Cl and KOH. In addition to the mesopores of *ca.* 3.71 nm determined by BET measurements (Table 1) for the HBFC-1, macropores with much larger pore size can be seen from the SEM image shown in Fig. 1a, which builds up a hierarchical porosity composed of mesopores connected with macropores/micropores. The hierarchical pore structure is very important to improve the electrical performance of the materials.^38,39^ The macropores/mesopores can serve as solution buffering reservoir to minimize the diffusion distance, facilitating mass transport, and also reduce the volume change during the charge/discharge cycling, ensuring a high cycling performance. While, the mesoporous/microporous make to increase the specific surface area and provide abundant adsorption sites for electrolyte ions. Besides, mesopores shorter diffusion pathways, thus improving the electrical performance of the materials.^40,41^ Apparently, in the case of the HBFC-2 sample where without adding NH_4_Cl at the carbonization process, it is observed to be highly accurate and less porous but approximates the surface from the porous due to activation by KOH as shown in Fig. 1b. The HBFC-3 sample with adding the blowing agent (NH_4_Cl) during of synthesis process, but without adding an activation agent (KOH) have a coherent three-dimensional (3D) structure of regular carbon chips is obtained but with less porous due to the non-activation as shown in Fig. 1c. The morphology of HBFC-4 without the addition of both (NH_4_Cl and KOH) maintained the microstructures, but it has a dense common blocky structure and has a smooth surface, as shown in Fig. 1d. Furthermore, the transmission electron microscope (TEM) was used to characterize the morphology of HBFC-1. The TEM image shows the HBFC-1 highly porous nanosheets structure with some structural defects as shown in Fig. 1e, and the HBFC-1 surface in the high-resolution was mesopores and micropore due to KOH activation, in Fig. 1f. This structure would provide an important accommodation region for electrolyte ions and charge X-ray diffraction (XRD) spectra, Raman spectrum and N_2_ adsorption and desorption were also employed to investigate the structure of the HBFCs, as shown in Fig. 2. From Fig. 2a, the patterns of four samples all display two broad diffraction peaks at 2*θ* = 23° and 43°, which are assigned to (002) and (100) lattice planes of disordered and graphitic carbon, respectively.

**Table 1:** Elemental Analysis, BET surface area, and pore structure characterization parameters of the different samples^a^

Sample	C%	N%	O%	*S*_BET_ (m^2^ g^−1^)	*S*_mic_ (m^2^ g^−1^)	*d* (nm)	*V*_total_ (cm^3^ g^−1^)
HBFC-1	85.10	1.71	13.19	1720.46	168.83	3.71	0.863
HBFC-2	57.42	1.00	41.58	1711.34	160.73	3.33	1.050
HBFC-3	—	—	—	377.57	30.41	3.51	0.198
HBFC-4	—	—	—	23.54	15.67	3.72	0.047

a where: *S*_BET_ ≡ specific surface area determined according to the BET method, *S*_mic_ ≡ micropore surface area from the *t*-plot method. *d* ≡ adsorption average pore diameter. *V*_total_ ≡ total pore volume.

**Fig. 3:**
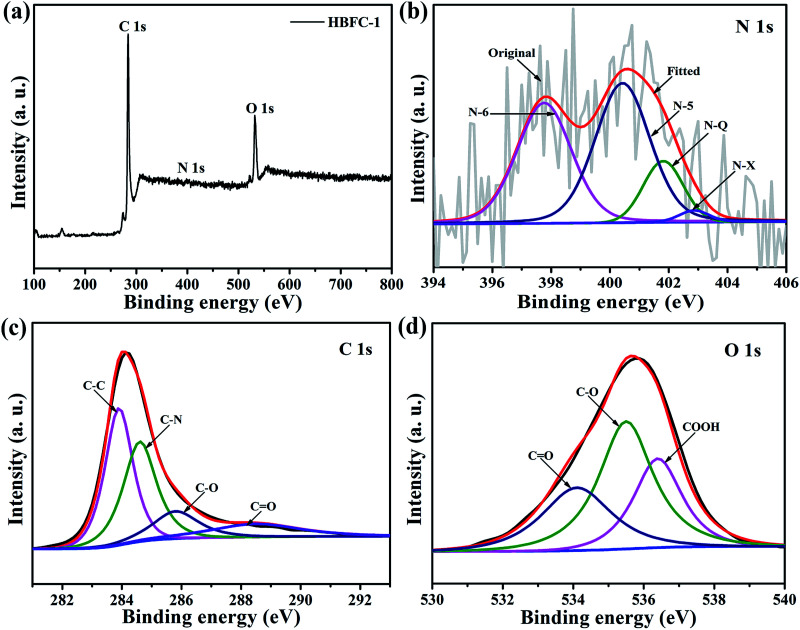
XPS spectra of the HBFC-1 nanosheet, (a) survey spectrum; (b) N 1s spectrum; (c) C 1s spectrum; and (d) O 1s spectrum.

**Fig. 4:**
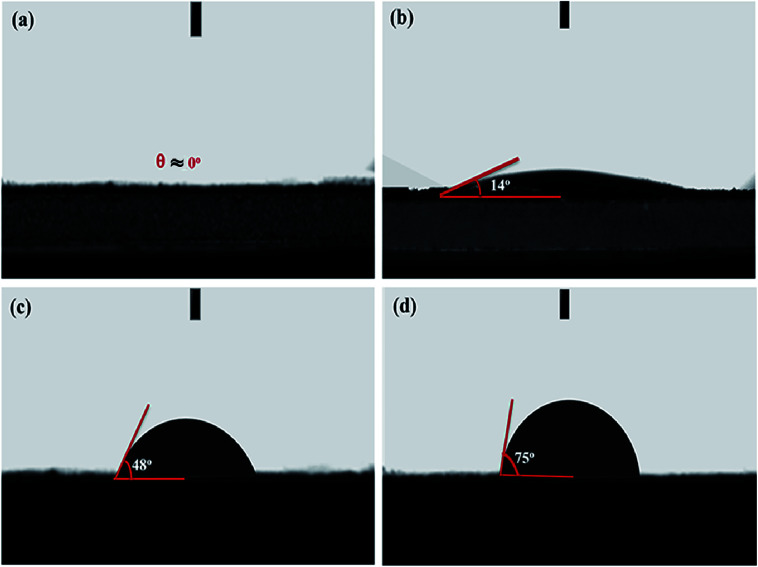
Optical images of water droplet on the surface of (a) HBFC-1; (b) HBFC-2; (c) HBFC-3; and (d) HBFC-4.

Particularly, the (002) and (100) peak of HBFC-1 and HBFC-2 shifts to lower angle as compared with HBFC-3 and HBFC-4 samples, suggesting the larger interlayer distance of HBFC-1, HBFC-2 samples due to the much thinner carbon nanosheets.^42^ Moreover, the (002) diffraction peaks can be distinguished in HBFC-1, due to the strong etching effect on the lamellar crystal structure by activation. This suggests that a disturbed structure in amorphous carbons is caused due to the randomly oriented hexagonal carbon layers, which is beneficial for increasing the specific surface area. To measure the quality of the active carbon and the materials dependent on it, we used the Raman spectrum as shown in Fig. 2b. It was noticed that all four samples have intense peaks located in the peaks of the active carbon about at 1340 cm^−1^ (D band) and 1590 cm^−1^ (G band), The *I*_D_/*I*_G_ value of HBFC-1 about 0.98, smaller than those of HBFC-2 (1.07), HBFC-3 (1.02) and HBFC-4 (1.00), which indicates the highest graphitization of HBFC-1 sample and pointing the formation of long-range graphitized carbon of HBFC-1 sample.^43^ The N_2_ adsorption–desorption isotherms of HBFC-1and HBFC-2 of type IV, while HBFC-3 and HBFC-4 are ty-pical characteristic of the type-I in Fig. 3a. The reason for the different N_2_ adsorption–desorption isotherms is that both HBFC-1 and 2 are added with KOH, while HBFC-3 and 4 are not added with KOH. KOH as an activator has a significant effect on pore development. Increasing the carbonization temperature to 800 °C increases the release of volatiles in the precursor, thereby increasing the development of pores and creating new pores. It can be seen that the adsorbed volume of HBFC-1 is noteworthy larger than that of HBFC-2, HBFC-3, and HBFC-4 samples, indicating carbonized of HBFC-1 materials by both KOH and NH_4_Cl activation can improve the porosity of carbon. Moreover, the HBFC-1 has an unambiguous hysteresis loop of (*P*/*P*_0_ ∼ 0.45–1.0), indicating the presence of several mesopores and macroporous structures. The detailed parameters for HBFCs are summarized in Table 1. It is noted that the specific surface area of the HBFC-1 sample is measured as ∼1720 m^2^ g^−1^, higher than that of HBFC-2 (∼1711 m^2^ g^−1^), HBFC-3 (∼378 m^2^ g^−1^), and HBFC-4 sample (∼24 m^2^ g^−1^). In contrast, the pore size distribution curve Fig. 2d shows that all samples have a major pore size distribution of mesopores, but the HBFC-1 has the highest pore size and volume, this may be for the use of NH_4_Cl and KOH in the carbonization process. Besides, as for the HBFC-4 sample, the pore size was the smallest, which indicates that without adding (KOH, NH_4_Cl) in the carbonization process. The higher specific area, pore size, and volume are a benefit for specific capacitance in supercapacitor applications.^44^ The binding energy and oxidation states of elements of HBFC-1 were verified by X-ray photoelectric spectroscopy (XPS) analysis, spectra (C 1s, N 1s, and O 1s) corresponding peaks are at about 284.2, 400.7, and 532.8 eV, respectively shown in Fig. 3a. The feature peaks of C, N, and O for HBFC-1 is calculated from the (XPS) survey spectrum to be about 85.10%, 1.71%, and 13.19%, and for HBFC-2 is about 57.42%, 1.00%, and 41.58% respectively, the percentage of N in HBFC-1 bigger than HBFC-2 due to adding the activation agent (NH_4_Cl), Table 1. An XPS analysis of HBFC-1 was performed, and the enlarged peaks of N 1s were treated with appropriate curves, wherein the high resolution of the N 1s spectrum demonstrated the presence of four nitrogen main types for HBFC-1,^45^ As shown in Fig. 3b, the peak at 397.7 eV corresponds to pyridinic N (N-6, N in 6-member ring) species, the peak at 400.5 eV shows largest attributed proportion to pyrrolic structure (N-5, N in 5-member ring), the peak at 402.9 eV for pyridine-N-oxide (N-X), and the peak at 401.7 eV is due to graphitic-N (quaternary-N) (N-Q).^46^ The results indicate an increase of nitrogen ratio in the carbon structure for HBFC-1, due to the activation agent containing nitrogen (NH_4_Cl). As shown in Fig. 3c, the high-resolution C 1s spectrum can be removed from the HBFC-1 sample as-prepared for four peaks that can be attributed to the following carbon types: C–C (graphitic carbon), C–N, C–O and O–C=O are a single component at the following corresponding peaks 284.0 eV, 284.7 eV, 285.9 eV, and 288.5 eV, respectively.^47^ The peak (284.7 eV) corresponding C–N, can also come from C–C of sp^3^ hybridized carbon. Also, we find that in the same way, O 1s can suit three distinct peaks that can correspond to the presence of surface oxygen groups in the form of C=O (Quinone groups), C–O, and COOH, corresponding peaks at about 534.1 eV, 535.5 eV, 536.3 eV, respectively, as shown in Fig. 3d.^48^ The presence of combined functional groups can work in the structure of the activated porous carbons derived from HBFC-1 as active sites for individual reactions during the discharge process, and due to reduced oxidation, functional groups rich in nitrogen and oxygen atoms can provide the advantage of false capacitance during the discharge process. These results indicate that the activated carbons by (NH_4_Cl and KOH) derived from HBFs have a large specific area and are distinguished in achieving high electrochemical performance when used in energy storage devices. Water contact angles evaluated surfaces wettability of HBFC-1, HBFC-2, HBFC-3 and HBFC-4 samples. The optical images of water droplets on the surfaces of the carbon layers at a time about 0.2 s are shown in Fig. 4. The contact angle (*θ*) for HBFC-1 is 0°, as shown in Fig. 4a, this reveals the super hydrophilicity after activation of HBFC-1. The enhanced hydrophilicity of HBFC-1 is mainly derived from the high content of nitrogen and oxygen on the HBFC-1 surface, which can reinforce the interaction forces toward aqueous electrolytes through polar attraction and hydrogen bonds. As for the HBFC-2 sample has a contact angle (bigger than 14°), as shown in Fig. 4b, also the HBFC-3 sample has a relatively large contact angle (48°) due there is without activating agent (KOH), as shown in Fig. 4c, and we find that the HBFC-4 sample has a large contact angle (75°) which indicates that without adding both activations (KOH, NH_4_Cl) for carbonization process, standard for wettability between liquids and solid materials and for measuring wettability on the surface treatment of various reagents.

### 3.3. The electrochemical performance

**Fig. 5:**
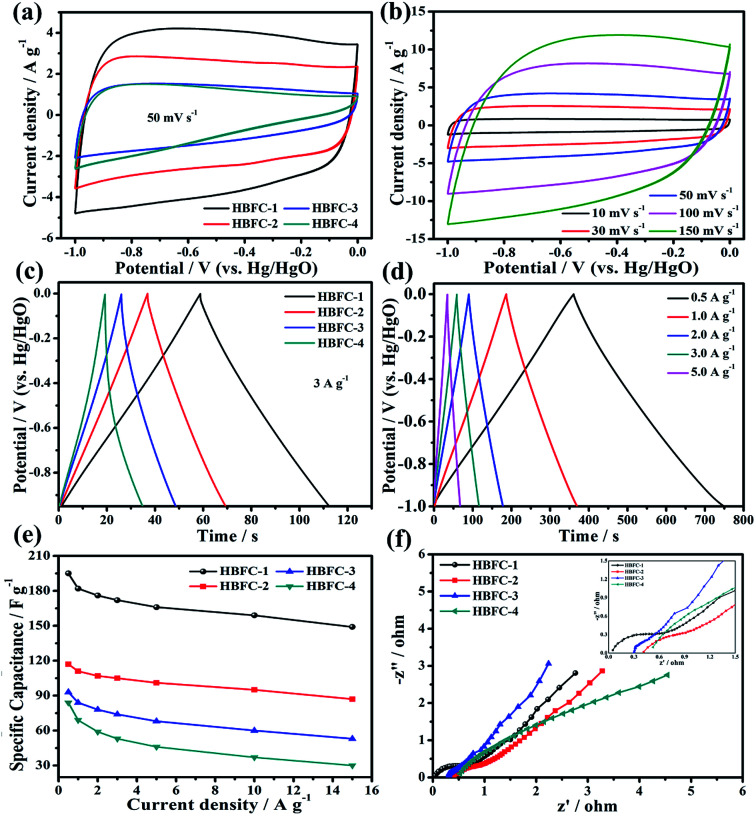
(a) CV curves of HBFC-1, HBFC-2, HBFC-3, and HBFC-4 at 50 mV s^−1^; (b) CV curves of HBFC-1; (c) GCD curves of HBFC-1, HBFC-2, HBFC-3, and HBFC-4 at 3 A g^−1^; (d) curves of the GCD curves of HBFC-1 at different current densities; (e) specific capacities of the HBFC-1, HBFC-2, HBFC-3, and HBFC-4 electrodes at different current densities; (f) Nyquist plots of the HBFC-1, HBFC-2, HBFC-3, and HBFC-4.

To analyze the electrochemical performance results of the HBFCs prepared, three-electrode configurations were first made in 2 M KOH aqueous solution; the results are illustrated as shown in Fig. 5. The cyclic voltammetry (CV) curves of the HBFC-1 electrode, as shown in Fig. 5b, exhibit a rectangular shape, with varying scan rates ranging from (10 mV s^−1^ to 150 mV s^−1^). As to its CV curves at 50 mV s^−1^ of the four samples, the HBFC-1 electrode has the highest current density and the desired rectangular shape, CV curves of the HBFC-3 electrode as well as the triangle HBFC-4 electrode, this characteristic leads to undesirable poor capacitive behavior, as shown in Fig. 5a. The GCD curve with a quasi-triangle character, as shown in Fig. 5d, and all the symmetrical curves imply excellent electrochemical reversibility of HBFC-1.^49^ As shown by the curves at the current density of 3 A g^−1^, as shown in Fig. 5c, the discharge time of the other three samples is much shorter than the HBFC-1 electrode, which has a relatively long discharge time, indicating a higher double-layer capacity. Then, the HBFC-1 electrode shows significantly higher specific capacities at a current density of 1 to 10 A g^−1^, as shown in Fig. 5e. Specifically, the order of capacities HBFC-1 is as follows: of 195, 182, 176, 172, 166, 159, and 149 F g^−1^ at the current densities of 0.5, 1, 2, 3, 5, 10, and 15 A g^−1^, respectively.^50^ To evaluate the electrochemical properties, electrochemical impedance spectroscopy (EIS) test is performed for the four samples (Fig. 5f). Note that all Nyquist plots have a linear slope in the low-frequency region. Besides, Nyquist plots of HBFC-1 have a small semicircle in the high-frequency region (inset left in Fig. 5f). The doping of N in carbon usually enhances electrochemical performance compared to HBFC-2, HBFC-3, and HBFC-4 samples.^51^ Therefore, we conclude the factors that improve the specific capacity of the HBFC-1 sample, where we find that the addition of the chemical blowing agent (NH_4_Cl), which in turn increases the nitrogen content in the HBFC-1 sample and that the HBFC-1 has a graphene-like structure and the presence of mesopores abundant, make ion transfer fast. The HBFC-1 sample provides a large specific surface area that makes it have more electrochemical active sites.

**Fig. 6:**
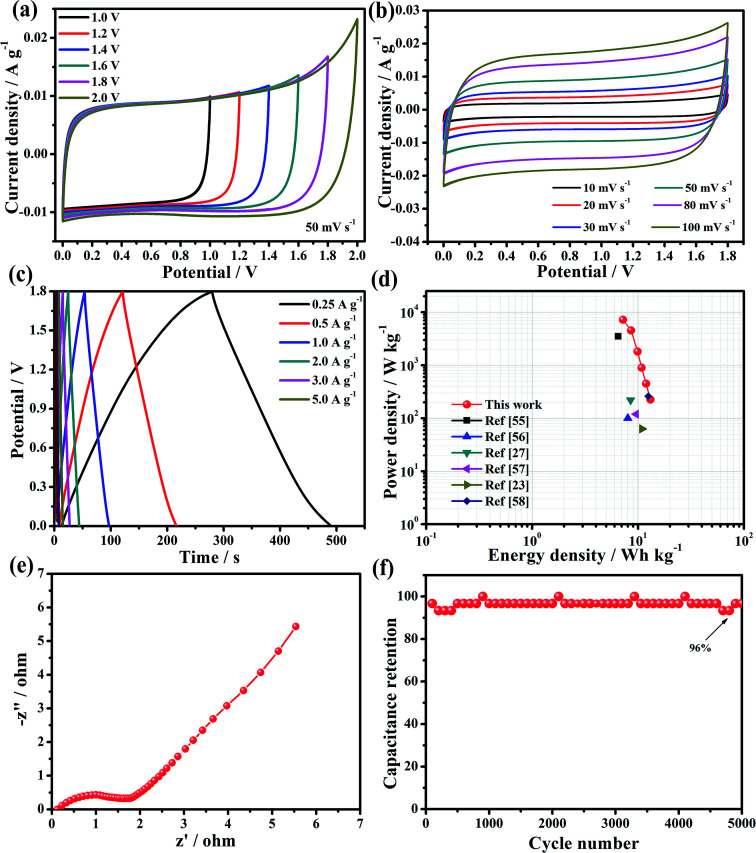
(a) CV curves of the HBFC-1 symmetric two-electrode cell (50 mV s^−1^) at different voltage windows in 0.5 M Na_2_SO_4_ aqueous electrolytes; (b) CV curves of the SSC at different scan rates; (c) GCD curves of the SSC at various current densities; (d) Ragone plots of the HBFC-1//HBFC-1 device in comparison with other biomass-derived, carbon-based supercapacitors; (e) Nyquist plots of the HBFC-1 symmetric supercapacitor and (f) cycling stability of the HBFC-1 symmetric supercapacitor.

For the fabrication of the two-electrode symmetric supercapacitor and to learn more about the electrochemical performance of the HBFC-1 electrode-based supercapacitor, we find that the HBFC-1 symmetric supercapacitor (HBFC-1//HBFC-1 SSC) is assembled in 0.5 M Na_2_SO_4_ electrolyte. To confirm the electrochemical stability and operation of the voltage range of the device, first, the symmetric supercapacitor was measured in a different suitable range from 1.0 V to 2.0 V, as shown in Fig. 6a. It is noticeable that the CV of the HBFC-1//HBFC-1 SSC remains in the form of an ideal rectangular of 1.0 V to 1.8 V, so we chose the potential 1.8 V because it has the ideal behavior of large capacity. However, if we look at Fig. 6a, the continuous increase in voltage of more than 1.8 V, for example, 2.0 V, changes the ideal rectangular shape to increases sharply.^52^ Therefore, an operating voltage of 1.8 V was selected for the following electrochemical performance test for CVs. Typically, CV curves of the HBFC-1//HBFC-1 SSC at potential (1.8 V) were made at different scan rates ranging from 10 to 100 mV s^−1^ as shown in Fig. 6b. We note that the shape of the CV curves does not change significantly even at the highest scan rate; this indicates the good rate ability and ions' rapid transition. The GCD curves of the symmetric supercapacitor at different voltage range from 1.0 V to 1.8 V, as in HBFCs, the GCD curves have optimal electrochemical reflection potential.^53^ Also, an operating voltage of 1.8 V was selected for the following electrochemical performance test of the GCD curves. Therefore, the GCD curves of the HBFC-1//HBFC-1 SSC, which range from (0.25 to 10 A g^−1^) at 1.8 V as shown in Fig. 6c and the specific capacities of the HBFC-1//HBFC-1 SSC are as follows of 29, 26, 24, 22, 20, 19, and 16 F g^−1^ at the current densities of 0.25, 0.5, 1, 2, 3, 5, and 8 A g^−1^, indicate that the discharge curves are very symmetric with their corresponding parallel and also the charge and discharge curves are symmetric with the change of time, which indicates that HBFC-1//HBFC-1 SSC has high electrochemical reversibility. As shown in Fig. 6e, the Nyquist plots for HBFC-1//HBFC-1 SSC provide a semicircle line in the high-frequency region which can be due to interfacial charge resistance, and the vertical line has ideal capacitive behavior at the low-frequency level and represents the frequency region Warburg resistance is low and associated with fast ion transfer between electrode and electrolyte.^54^ This result can be suggesting to the porous interconnected carbon nanosheets structure and the increase in nitrogen content resulting from the addition of a chemical blowing agent to the HBFC-1 material, so the plots indicate the favorable capacitance characteristic of the supercapacitor. The cycle stability of HBFC-1//HBFC-1 supercapacitor at 3 A g^−1^, the capacitance retention is high as 96% after 5000 cycles, indicating the excellent cycling stability of HBFC-1//HBFC-1 SSC, as shown in Fig. 6f. The Ragone plots are the main objective of linking energy and power density to affect high-performance supercapacitors positively. When the power density is 225 W kg^−1^, the maximum power density is 13.1 W h kg^−1^ and goes down to 6.67 W h kg^−1^ slightly while the power density increases to 8893 W kg^−1^, as shown in Fig. 6d and Table 2. At high discharge rates, the device offers the highest energy density of this work compared with other biomass reports and carbon-based symmetric supercapacitors.^23,27,55–58^ The previously selected voltage range (1.8 V) improves the supercapacitor's power density according to equal equ. *E* = 1/2*CV*^2^. For an explanation of these results, we note that such high-efficiency carbon material for the supercapacitor electrode is derived from renewable materials as abundant hibiscus fruits through the use of NH_4_Cl as an effective blowing agent and KOH as an activation agent.

**Table 2:** Comparison of electrochemical performance of symmetric cells in the references

Sample	Energy density (W h kg^−1^)	Power density (W kg^−1^)	References
FHPC	6.5	3500	55
GNCs	8.0	100	56
ACs	8.5	220	27
MCSF	9.6	119	57
ABCs	10.9	63	23
LSC800	12.5	260	58
HBFC-1	13.1	225	This work

## 4. Conclusion

In summary, the prepared graphene-like carbon nanosheets (HBFC-1) derived from the biomass of the hibiscus sabdariffa fruits in which using ammonium chloride is used to decompose within the sample give ammonia and hydrogen chloride gases, which blow off and detonate some carbon in the aerogel phase. We find that carbon nanosheets in the (HBFC-1) sample make an effective bond with KOH (activation agent), which showed as a result that the HBFC-1 sample has short paths for transmission ions because they contain active electrochemical surfaces. Therefore, the HBFC-1 electrode gives high specific capacitance. Using it as an electrode, the symmetrical supercapacitor (HBFC-1//HBFC-1 SSC) was assembled in a 0.5 M Na_2_SO_4_ solution. The symmetrical supercapacitor has a high specified capacity (29 F g^−1^ at 0.25 A g^−1^) and a high energy density of 13.1 W h kg^−1^ at a power density of 225.00 W kg^−1^. After 5000 cycles, the initial capacitance still retains 96%. Its energy density and power density are much higher than other similar supercapacitors assembled by biomass activated carbon. Besides, the work of the material of plant (Hibiscus fruits) preparing a graphene-like carbon material of good porosity, and further applied to an energy storage system having a general significance.

## Conflicts of interest

There are no conflicts to declare.

## Supplementary Material
